# Clinical Review and Hospital Reports

**Published:** 1836-01-01

**Authors:** 


### Clinical Review.

### County of Meath Infirmary.

The more we see of our Dublin contemporary, the more we are disposed to admire the spirit with which it is conducted. It is impossible to read its successive numbers without being struck with the general value of its articles, and the professional attainments of its contributors. We are delighted to observe this—we are delighted beyond measure to see in Ireland, as elsewhere, how great has been the progress of medical science, how much greater its progress must be when such energy is employed in its advancement. Success we need not wish to our contemporary, for success it has obtained already; but we do sincerely wish it a continuance of those exertions on the parts of all concerned in it, which have placed the laurels already on its brow, and will ever keep them green.

In the management of a periodical medical journal there are two plans open to its conductors. They may either select extraordinary cases, or captivating speculations, and, ever on the hunt for something new, they may tickle the tastes of their readers by this species of diablerie—or, pursuing a more direct and more quiet path, they may point attention to what seems likely to be useful and true. Before men had discarded the subtleties of the Aristotelian philosophy, and seen in induction the objects and the scope of knowledge, simplicity and science were considered incompatible. System after system, fanciful and brilliant, rose like a rocket before men's eyes, and, just when it seemed at its zenith, broke. It broke, but another rocket blazed, and some fire-work was always in the clouds. In the midst of these false lights, inductive reasoning like the day was drawing on, and when it fairly dawned how yellow and how dull those artificial fires became. We have long professed ourselves the prophets, or rather the followers of the new philosophy—the zealous, though the humble instruments of disseminating its beneficial truths. We have been called, and we are, utilitarians in medicine, and we would rather confirm in the minds of doubting readers one substantial fact, than parade before them the most shewy array of critical or speculative abstractions.

We have usually chosen for extract, analysis, or criticism, papers, which wear what we consider a practical tendency. But what are practical facts, and who are practical men? That is just the point on which we would offer a few remarks.

The persons in our profession who term themselves practical men, are too often exceedingly shallow fellows. They are men with little learning, and with much prejudice—the alluvies left by the subsidence of the old Hippocratic and empiric systems. They are, as Tristam Shandy has it, for the most part very hobby-horsical. They have great fancies for particular remedies—for particular conditions of system—talk much of idiosyncrasies—are fond of the good old terms, asthma, and white-swelling, and so forth. These gentlemen usually exclaim against the mania for morbid anatomy. If we were asked the diagnostic marks of such a practical man as we have drawn, we should say, his dislike, more or less avowed, of morbid anatomy, and his being always prepared with some remedy to knock some particular complaint on the head.

The true practical man is of a very different calibre. He is one who is thoroughly imbued with the spirit of inductive reasoning—who sees in anatomy, healthy and morbid, the elements of all the exactness we can give to medicine—who studies symptoms in reference to such anatomy—and who simply tests the powers of remedies by simple experiments. Men of this description are becoming, we are happy to say, more common. Among the well-educated members of our profession, they are daily met with. They are the symbols of the new order of things, for a new order of things there is. Such, as Iago says, we do profess ourselves, and to make more and more men in the profession such, we have worked, and, please God, we will work still.

This brings us to the point we aimed at. Is practical information of the description we have mentioned to be obtained by indulgence in disquisitions on abstractions—or in the plain study of plain facts? Suppose a man wishes to become practically conversant with surgery. If he possesses good sound sense, he does not lock himself up in his chamber, and devote night and day to the reading of Rhazes, and Albucasis, and Celsus, and Paré, and Benjamin Bell. No, he goes to the dissecting-room, and he learns anatomy thoroughly, and he also learns the elements of surgery and physic and materia medica, and being thus acquainted with the leading and established facts, he proceeds to the hospital ward and the hospital dead-house. He accurately notes the cases that present themselves, and the symptoms they exhibit. If the patient dies, he reads in the dead body the language of symptoms—if he recovers, he has observed the effects of medicine. This is the course of study which turns out a good surgeon.—Journals should be conducted with a view to the same substantial results. They should collect the facts too widely scattered for individual observation—and they should present those facts, those simple facts, for general perusal and consideration. This is our notion of what a practical journal should attempt, and for these reasons we usually select those cases which can be resolved into useful lessons for the man of much or of little experience. We leave to others the insatiate thirst for novelty, which sometimes gratifies but too often disappoints, and we content ourselves with endeavouring to point out what we think is sound philosophy—close observation, and rigorous reasoning.

The paper we are now to notice is by Dr. Browne, one of the surgeons of Saint Mark's Hospital, and formerly surgeon to the County of Meath Infirmary. It is offered with a view to the elucidation of the surgical pathology and treatment of aneurism. He observes that in both parts of this subject there is still much difference of opinion, and he presents the following facts in order that they may tend, so far as they can, to increase our stock of knowledge. The cases are four in number, and occupy twenty-eight pages of our contemporary. They admit, we trust, of much compression.

CASE 1. *Aneurism of the Posterior Tibial Artery—Scarpa's Operation—Death from Sinuses in the course of the Sartorius Muscle.*

A man, æt. 27, admitted into the Infirmary, Aug. 27th, 1817. On examination, there was found an aneurism of the posterior tibial artery. A round tumor, resembling an orange in size and shape, existed in the centre of the upper and back part of the calf. It was deep seated, lying apparently beneath or in front of the belly of the gastrocnemius muscle; its upper edge was placed nearly three inches inferior to the lower margin of the popliteal space, and it extended downwards and laterally more than two inches in each direction. Pressure on the femoral and popliteal arteries arrested the pulsation, and diminished the size of this tumor. The disease had existed for two months. There was no disturbance of the health, of any consequence.

On the 1st of September, Dr. Browne performed the operation of tying the femoral artery, immediately above the sartorius. Some difficulty was experienced in finding the superior border of that muscle, and it was necessary to turn it outwards. Two ligatures were applied to the artery, on tying which the pulsation in the tumor ceased.

On the 2d day there was such symptomatic fever that twenty ounces of blood were abstracted, and purgatives, &c. were given. On the 3d day the bleeding was repeated. On the 10th day, the lower ligature separated, the upper having come away on the 9th. Slight lividity of the integuments covering the outer ankle, and superficial ulceration between the toes now occurred. The aneurismal tumor was reduced to a small hard substance of the size of half a nutmeg. An abscess now formed in the course of the artery upwards, and a sinus communicated with the wound. The patient got hectic with diarrhœa. On the 36th day an opening was made in the groin. The probe was now found to pass nearly its entire length on the inner side of the sartorius muscle, and deep in the flesh of the thigh; the discharge was very considerable. The hectic symptoms increased, and on the 41st day, the patient died, having previously evinced some features of thoracic inflammation.

Dr. Browne's remarks may be easily summed up. He inquires into the cause of the fatal sinuses beneath the sartorius. He thinks that they depended on an original want of vis vitæ—and he furthermore thinks that the patient's death was occasioned by an aortic aneurism. The reasons for the latter extraordinary conclusion are, that the patient had a vibratory pulse, and that he had pain under the sternum with lividity of countenance the day before he died.

The latter supposition is so unsupported, that it is not worth while arguing it. But we would make this one observation on the cause of the collections of matter. We are not surprised at their occurrence, considering that the muscle was a good deal disturbed during the operation, and that the patient lost forty ounces of blood from the arm within two or three days after it. The two circumstances taken together will present a tolerably satisfactory ground of conjecture to most practical surgeons.

Case 2. *Diffused false Aneurism of the Popliteal Artery, from Wound by the Sequestrum of a necrosed Femur—Operation—Death.*

A young man, æt. 25, who had laboured under necrosis of the lower third of the right femur for some years, and in whom two fistulous openings discharged moderately, one on each side of the thigh a little above the joint, was induced to dance at a wedding. In the midst of the glee, he felt sudden uneasiness in the thigh, and bleeding took place from each fistulous opening. Some compression was resorted to, but more or less hæmorrhage occurred for eight days, when he was received in the Meath Infirmary, under the care of Dr. Byron.

This gentleman finding that compression on the femoral artery arrested the bleeding, with great promptitude, decided upon tying that vessel in the upper third of the thigh; which operation was performed at the moment, without difficulty, and with the effect of putting an immediate stop to the hæmorrhage. The *vis vitæ*, however, had been previously too far exhausted to allow of salutary reaction, and the limb passed rapidly into gangrene, which quickly extended to the groin, and carried him off in a few hours.

The *post mortem* examination discovered a longitudinal slit nearly one-fourth of an inch long, in the front of the poplitæal artery, close to which lay a jagged portion of sequestrum; this was thin and sharp, and formed a part of half of the circumference of the cylinder of the old bone, the greater portion of which was firmly incased in the new one.

Dr. Browne refers to two similar cases, one related by Dr. Jacob, and the other by Mr. Porter. In Dr. Jacob's case, amputation was performed, and the patient recovered. In Mr. Porter's the operation could not be performed, and the patient died. From various considerations, Dr. Brown considers amputation preferable to the ligature of the femoral artery in such cases, and most surgeons, we think, will feel inclined to agree with him.

Case 3.—*Circumscribed false Aneurism of the Brachial Artery; a high Bifurcation; both Branches tied; the Aneurismal Sac opened; Recovery.*

P. G. æt. 32, a shoemaker, admitted into the Infirmary, Dec. 28, 1824. Four days previously he had forcibly wounded the brachial artery with his knife. Great bleeding occurred at the time, but was stopped by powerful compression. In forty-eight hours it recurred.

"On examination he was found to have received an incised wound on the inner side of the arm, two inches above the inner condyle, and one inch superior to the bend of the joint. This wound was an inch and a half long, and extended obliquely upwards and outwards; its surface was foul, but partially united, and its edges had been closed by four continuous sutures. To the outside of the wound was situated an aneurism half the size of a pigeon's egg, pulsating strongly and synchronously with the artery at the wrist; and the arm and forearm were swelled to at least twice their usual size, particularly on the fore and inner parts."

By appropriate means the wound was healed, but the aneurism remained. Gentle pressure on it was tried. It caused such irritation of the skin that the latter sloughed, and on the separation of the slough, hæmorrhage ensued. The following was the condition of the parts. The cavity of the aneurismal sac was exposed by an opening the size of a shilling, the sac itself being apparently as large as a common sized poppy head; there was considerable hardness and thickening of parts in the line of the brachial artery, extending up the arm for one hand's breadth above the sac; no pulsation was discoverable at the wrist, and the limb retained its natural temperature. The brachial artery was now tied above the tumor. Hæmorrhage issued from the latter on slackening the tourniquet; there was a high bifurcation. The second arterial trunk was tied; this did not stop the hæmorrhage. The following particulars are deserving of extraction without mutilation.

"The discharge of arterial blood having been altogether uninfluenced by these measures, it was determined to incise below the sac, and endeavour to tie one or both branches of the bifurcation, as might be found necessary. Accordingly, an oblique incision was made on the inner side of the arm, immediately below the sac, carried outwards two inches, and subsequently made angular, by giving it a direction towards the biceps tendon. The veins and nerves were exposed, and the artery sought for without success, and in the course of these efforts the sac was opened below.

During the continuance of the recent operation, the tourniquet had been occasionally loosened, as the size of the opening in the sac enabled the thumb, assisted by a compress of lint, to command the bleeding; it was then, however, tightened, and the finger passed into the sac. That cavity was found to be of an extent considerably larger than had been supposed; it extended two inches in the direction of the external hardness, and three inches to the outside of the biceps tendon; altogether it might contain four ounces of coagula.

As the sac had been exposed, and the second opening made into it, and as there seemed no prospect of finding the wounded vessel below, it was deemed right to throw both wounds into one by a suitable incision. This having been done, the brachial artery was discovered lying at the bottom and middle part of the sac, and in the centre was an opening or slit, half an inch in length, extending longitudinally, from which blood issued with the same freedom as before on the tourniquet being relaxed.

The artery was considerably enlarged, nearly to twice the usual size, and its coats much thickened, particularly superior to the opening which had been made into it; inferior to this aperture its condition was more healthy, and one inch below it its state was altogether normal. At that point, therefore, (a little dissection being previously required to bring it into view,) a double ligature was applied upon it.

This measure, though it materially checked, did not arrest the hæmorrhage, for an oozing of florid blood still continued from the opening in the artery on relaxing the tourniquet. It was deemed prudent, therefore, to include the thickened portion of the vessel lying in the sac in a ligature, which was accordingly done, two inches superior to the opening in it, and then only did the bleeding cease.

The sac did not extend quite an inch below the wound in the artery, although it reached more than three inches above it; its orifice was lightly filled with charpie, and the other wounds were brought together by adhesive plaster. Compresses of lint and tow, with a flannel roller, were applied over all, after which he was put to bed, and got thirty drops of tincture of opium, being directed to keep quiet and live low."

After this all went on well. On the fifth day, the surface of the exposed sac was granulating. On the eighth day there was feeble pulsation in the right radial artery at the wrist. On the 12th day all the ligatures came away; the cavity of the sac appeared obliterated. On the 15th day, slight pulsation in the right ulnar artery. On the 43rd day, the wounds were all healed, and on the 47th day the patient was dismissed. He then possessed nearly the full use of the limb. A strong pulsation was discovered in the profunda superior artery.

This is undoubtedly an interesting case. In aneurism at the bend of the arm, from puncture of the brachial artery, or of the radial or the ulnar, supposing a high bifurcation, two operations are open to the surgeon. Either he may perform the Hunterian operation for aneurism, and tie the artery above the tumor, or he may adopt the old operation—cut into the sac, expose the orifice of the vessels, and secure the latter above it and below it. Some authorities are in favour of the one, some advocate the other operation. We conceive that the rule of practice is clear. If pressure on the brachial artery, or on either or both of its branches, in the case of a high bifurcation, satisfactorily arrests the pulsation in the trunk, the surgeon should give his patient the chance of the ligature of the vessel in the arm. We have the experience of Mr. Colles in favour of this rational and moderate practice. With him, it has been highly successful. If, however, pressure in the artery in the arm does not sufficiently control the pulsation, &c. in the aneurism, or if the operation we have mentioned fails, then the surgeon must cut into the aneurismal tumor, and proceed in the ancient manner.

The case before us was complicated. It is not impossible, nor, indeed, improbable, that if the operation of tying the artery in the arm had been performed, instead of using pressure, and producing an opening by sloughing into the aneurismal sac, it is not, we say, improbable, that a cure might have been effected. For the sac having partially sloughed, the case became analogous to one of wound, rather than to one of aneurism. In an aneurism, the simple retardation of the current of blood appears to be equal to effecting coagulation in the sac: but, in a wound, that would be quite insufficient to arrest the hæmorrhage from a large artery. We can, therefore, readily understand, that tying the artery above the tumor would cure traumatic aneurism, under ordinary circumstances, at the elbow, but would fail to cure it if the aneurismal sac was laid open. The case, we repeat, is one very worthy of the attention of practical surgeons.

Case 4.—*Aneurismal Varix at the Bend of the Arm—Cure by a Bandage.*

A healthy labourer was admitted into St. Mark's Hospital, March 29th, 1830, for aneurismal varix of the left arm.

" The median basilic vein was dilated to about the same extent as we observe it to be when ordinary venesection is going to be performed; and the pulsations of the artery beneath it, accompanied by the peculiar thrilling sensation, resembling the noise produced by the wheel of a watermill, were distinctly and strongly perceptible both to the ear and to the touch. This thrill extended for at least an inch all round the site of the original puncture, and could be removed by moderate pressure on the vein, without the pulse at the wrist being interrupted.

Slight hardness existed in the situation of the wound in the artery, particularly towards its outer side, accompanied by yellowness of the skin along the inside of the arm; and the forearm could not be flexed beyond a right angle, nor extended perfectly."

He had been bled six days previously.

The blood flowed "per saltum," but was easily checked. Next day there was much ecchymosis, and a small pulsating tumor was observed under the orifice. In three days the orifice healed.

Under the direction of Dr. Browne, a compress of lint, wet with lotio plumbi acetatis, was applied over the tumor, which was kept in its place by a roller, carried round the forearm and elbow; and some ordinary purgatives were directed. Digitalis was added afterwards. The compression and the latter remedy together effected so complete a cure, that, on the 12th of April, the patient was discharged cured. The arm was in a natural state, the vein of its usual size, and the artery felt beating beneath it, without increased strength in its pulsations, or any surrounding hardness.

We subjoin the following remarks.

"Aneurismal varix, at the bend of the arm, according to the opinions of the most approved authors, usually progresses in three several ways, viz. 1st, in the majority of instances the tumour, after attaining a certain moderate size, remains stationary, and, with ordinary care, and the avoidance of unusual exertion of the member, continues to be free from either danger or inconvenience during the rest of life. 2dly. Either from over-exertion or improper treatment, the cicatrix formed between the wounded artery and vein gives way, blood is effused into the intermediate and surrounding cellular tissue, and a varicose aneurism is eventually produced. 3dly. By careful and gradual compression, more particularly in young subjects, adhesive inflammation may be excited, by which the orifice of communication between the artery and vein becomes closed, the varicose swelling removed, and one or both of these vessels obliterated at the place; most usually the vein, but often both vein and artery.

Few surgical practitioners, however, are aware, I believe, of the fact, that an aneurismal varix may be radically and perfectly removed, all the tissues concerned in its production being restored to their original integrity, as occurred in the case which I have detailed.

But a termination so favourable must be deemed a rare event, and to account for it we must presume, that just so much lymph was effused as sufficed to agglutinate the lips of the wound in the artery, without being secreted in a quantity great enough to endanger the obliteration of its canal.

That the gentle pressure of the roller contributed to this effect is probable; but we must not forget that such an agent has been reprobated by authors, as giving rise, on several occasions, to the second termination of the disease which I have noticed, namely, the formation of a varicose aneurism; it should, therefore, be used cautiously, and its effects carefully watched."

The preceding cases merit the perusal of our readers.

---

### St. Thomas's Hospital.

### Syphilitic Eruptions treated with Hydriodate of Potass.

Our readers are aware that the experiment of treating several forms of syphilitic secondary symptoms, by preparations of iodine, has been lately made. We have noticed the observations on this subject that have been, from time to time, presented to the public, and we have watched, with some small degree of curiosity, the amount of faith placed in the medicine. In the reports of St. Thomas's Hospital, edited by Mr. South, we find five cases of secondary eruptions, treated with hydriodate of potass. These cases we shall notice separately.

Case 1.—*Roseola Syphilitica.*

"Joseph Lawrence, æt. 17, tailor, admitted June 19th, 1834. Of rather intemperate habits. States that, about eighteen months since, he had a chancre on the penis, and a bubo in each groin; for which he took mercury, and his mouth was kept sore for some weeks. Under this treatment, the chancre quickly healed and the buboes dispersed. About a month after the sore had healed he was attacked with sore throat, pains in his limbs, and an eruption of a pustular kind, appearing chiefly about the legs: in consequence of which he was admitted into the hospital, under Dr. Williams' care, and remained there for five months; during which time several pieces of bone came away from the nose and pharynx. He was treated with hydriodate of potass, and left the hospital quite well. About a fortnight or three weeks after, however, the pains in his limbs returned, the throat again became sore, the nose swollen, and a piece of bone in progress of separation, when he was re-admitted.

He complains of great pains in the limbs, which increase so much at night as to prevent his sleeping. His tonsils are red and swollen, and each has a large deep sloughy ulcer upon it, accompanied with slight superficial ulceration of the back of the pharynx. He has upon his back an indolent ulcer with a dark coppery-coloured margin, which is the remnant of an old pustule. The testicles are painful and tender. These symptoms are accompanied with slight constitutional fever; he is usually thirsty, has a furred tongue and bad appetite, and sometimes perspires much at night. *Ung. hyd. nitr. Oxid. gutturi applic.*

June 21. Much the same. *Garg. acid. mur.*

July 5. Throat much improved: the pains in the limbs much the same. An eruption of an exanthematous character has made its appearance, chiefly about the arms, breast, and insides of the thighs, consisting of patches, small, separate, and of various figure—not much unlike measles or scarlatina, in some places being somewhat elevated and accompanied with slight itching and smarting: it is of a hepatic brown colour, and becomes much more distinct on rubbing the part. ℞. *Pot. hydriod.* gr. viij. *ex Mist. camph. ter die.*

July 11. The pains are less severe. He has a considerable discharge of yellow mucus, tinged with blood, from the nose, for which he was ordered to inject *Lot. hydr. cin.*

July 15. Pains considerably diminished, and he now sleeps well at night. The eruption is not quite so fresh, but is still visible about the chest and arms. *Potass. hydr.* gr. x. *ter die.*

Under this treatment he continued improving, and by

Sept. 20. The throat was nearly well and the pains trifling.

Oct. 1. His throat is now well: the eruption has disappeared, and the pains in his limbs have entirely left him. But a little discharge from the nostrils continuing, he remained in the house till

Oct. 14, When he was discharged perfectly cured."

We could not beneficially abridge this case, on which we have some remarks to offer. We pray our readers to mark these particulars. A patient has an ulcer, for which his mouth is kept sore for "some weeks." In a month after the sore heals, he is attacked with sore throat and pustular eruption. He is treated with hydriodate of potass, and after *five months*, and the separation of pieces of bone from the pharynx and the nose, he is reported to be cured. Immediately after this recovery, he is again as bad as ever, and so he goes on for *eighteen* months, when he has sloughy ulcers of the throat, and a cachectic ulcer on the back. He is again in the Hospital for *four* months, again treated with hydriodate of potass, and again dismissed with the ticket of "cure."

We must say that this case carries any thing, to our minds, but evidence in favour of the hydriodate of potass. The symptoms were evidently of that mixed character, which results from the injudicious use of mercury in the first instance, in a patient whose constitution is impaired by intemperance. Such cases are usually tedious, and always troublesome, yet we venture to say that well-regulated courses of sarsaparilla, alternated with purgatives and with other tonics, and conjoined, now and then, with some mild form of mercury, as the hydrargyrus cum cretâ, or the oxymuriate, in very small doses, for ten days or a fortnight at a time—we venture to say that, after the lapse of *twenty-two months*, the patient would have been more permanently well than, in this case, he probably is.

CASE 2.—" *Purpura Syphilitica.*"

Isaac Gower, æt. 19, admitted Sept. 4, 1834. " He states that about six months ago he had gonorrhœa accompanied with chancre on the penis, but no buboes. He got well in a fortnight without mercury. Three weeks after, however, pains in the limbs supervened, affecting chiefly the knees, legs, and ankles, which were tender. An eruption broke out soon after, which continued more or less till his admission.

He now complains of severe pains in the limbs, more especially in the legs and large joints, aggravated at night so as to interfere with his rest. The periosteum on the tibia is tender, but not thickened. The eruption consists of small distinct specks, or patches, occupying chiefly the arms, breast, and legs, but especially the fore-arm. These spots are of a hepatic or dark coppery colour, of various size, from the most minute point to that of a flea-bite, and commonly circular. They are not accompanied with any visible elevation of the cuticle, nor are they attended with any itching. A few spots of a vesicular and pustular character are scattered about the chest, arms, and legs. He complains of slight sore throat, and the tonsils are rather swollen. He is somewhat feverish, being thirsty, and having his tongue slightly furred. ℞. *Tinct. Hyosciam.* ʒss. *ex Mist. Camph. ter die.*

*Sept.* 18.—The feverishness and sore throat have subsided, but in other respects he is much the same. ℞. *Potass. Hydriod.* gr. viij. *ex Mist Camph. ter die. Ol. Ricini* ℥ss. *p. r. n.*

*Sept.* 30.—Much improved. The pains have so much subsided that he is able to get a good night's rest. The spots are fading, their edges being not so well defined as before, and there is no fresh eruption.

*Oct.* 10.—Pains in the limbs entirely subsided. The eruption is nearly unaltered since the last report, but a few tubercular spots have appeared on the chest and arms.

He continued gradually improving without any change in the treatment up to.

*Oct.* 27.—When he was presented cured, a slight trace of discolouration only remaining in one or two places."

We do not exactly understand why this should be termed " purpura syphilitica." Purpura is a term which is applied to extravasations of blood in the cutis, and we presume there were none in this patient's case. But the name does not much signify, if the thing is understood. We prophecy that in this instance there will be a return of the symptoms ; we have not a doubt of that. The experiment is not worth a farthing ; for the patient should have been kept in the hospital, or if not kept in the hospital watched, for many months after the fading of the eruption. Patients with lichen and even with tubercle will, under salines and the most simple treatment, present quite as much amendment as this patient did. Nothing is more common. But then after a period other symptoms are developed—fresh ulcers in the throat—fresh eruption on the skin. We repeat that this case proves nothing.

The third case was one of " Lepra Syphilitica." The patient was in the house for a week, and left it " improving." This proves nothing neither.

CASE 4.—*Rupia Syphilitica.*

G. Toovey, æt. 27, an intemperate man, admitted Oct. 23, 1834, " states that about four years ago he had gonorrhœa and chancre on the penis, accompanied with bubo in each groin, which continued for some months, but subsequently were cured with mercury, his mouth having been made sore for a fortnight. With the exception of occasional pain in the left shoulder he continued apparently quite well till seventeen weeks since, when an eruption broke out on the legs, arms, and back, appearing at first like small vesicles, which gradually extended, breaking into large scabby sores, and afterwards healed, leaving the skin of a deep livid colour for some length of time. Other sores, however, quickly supervened. The eruption was accompanied with great pains in the limbs, more especially in the larger joints and shins, which were much aggravated at night. He has taken great variety of medicine for this affection, of which mercury formed the largest proportion, but without benefit.

When admitted he complained of severe pains in the limbs, especially in the legs, and also in the joints of the left wrist, knee, and great toe, which were rather tender. The periosteum of the left tibia is thickened, and extremely tender. The pains increased towards night, and prevented him from getting sleep. The eruption is principally about the legs, thighs, arms, and back: it consists of spots or patches of irregular incrustations, for the most part circular, and varying in size from that of a split pea to that of a half-crown. Some are of a conical shape, but more commonly, however, the scales are thin, superficial, and easily rubbed off, slightly inflamed at their base with a very narrow areola of a copper colour. Several blotches of a deep livid colour, the remains of old sores, are scattered over the body; but there is no appearance of the eruption in its primary or vesicular stage.

He has no sore throat, but says he has occasionally passed great quantities of blood by stool; at times, also, there has been a discharge of bloody mucus from the nose accompanied with pain, but the nose is neither swollen nor tender, nor have any pieces of bone come away from it.

His health is much broken up; he is very hot about night-time, and perspires largely towards morning: tongue coated with a whitish fur, and red at the tip: mouth still sore from the use of mercury. ℞. *Potass. Hydr.* gr. viij *ex Mist. Camph. ter die.*"

He gradually improved till the 20th of November, when the report states that he had scarcely any scabs, but several patches of a deep livid colour. The periosteum on the left tibia was still tender and painful. His health was much recruited. On the 25th of December he was discharged.

Nothing can be more deceptive than reports of this description. Patients labouring under rupia, or other cachectic symptoms, when received into a hospital and well clothed, well housed, and well fed, and, medically speaking, well attended to, almost invariably improve greatly. We have again and again seen patients admitted into the Lock Hospital with rupia, get rid of the scabs, and get fat and well under diet alone. But that is not a cure— far from it. The patients are dismissed apparently recovering fast or recovered. They generally have a relapse in a very short period. The treatment of rupia and the secondary symptoms allied to it, is one of the most interesting, and, at the same time, most complex problems in the management of the venereal disease. Of one thing we are certain from experience, that iodine does not cure it. So far as we have seen, iodine, in some of its forms, is of more service in this than in the other forms of secondary syphilis. We have to ring the changes on many remedies, while we steadily keep in view the improvement of the health. Iodine is one of the many. Small doses of mercury— steel—arsenic—iodine—all are of service in their turn. The *great* remedies are small doses of mercury and courses of sarsaparilla.

Case 5. *Syphilitic Lichen.*

John Cushion, ætat. 26, a seaman of intemperate habits, admitted Nov. 13, 1834. Had gonorrhœa and sores seven weeks before his admission. For these symptoms he took only salts. In a fortnight the primary symptoms were removed, but pains in the limbs, followed by eruption, came on.

When admitted a papular eruption was observed over the whole body, especially on the arms, thighs, legs, and chest—in some parts distinct, but in others in small clusters. Some had the appearance of small tubercles or acné: others, on the arms and chest, were more acuminated; while a few spots appeared obscurely vesicular. All were surrounded with a base of deep hepatic hue, and somewhat inflamed. He did not complain of itching, but there was a smarting sensation, or rather a heat in the part. He had severe pains in the limbs which prevented his sleeping: the right tibia was very painful, and the periosteum thickened and tender, more especially about the junction of the middle with the lower third of the bone. The left leg was much swollen and œdematous, and had an irritable ulcer both on the fore and back part, the result of a burn: both were somewhat swollen and tender. Both ulnæ were swelled and tender. And he also complained of tenderness over the whole skull. The left eye was dull, weak, and bloodshot; but there was not much intolerance of light, and little if any pain: the palpebral linings were rather moıe vascular than usual, and there was an increased secretion from the tarsal glands which were rather enlarged: the vessels of the ocular conjunctiva were injected with red blood, and the eye had a remarkably dull and muddy appearance: the pupillary margin was irregular, but the iris still retained its brilliancy. The tonsils were rather enlarged, and the scars of old ulcers very distinct. He was very feverish, complained of a burning heat over the whole body soon after getting into bed at night, which was followed by profuse perspiration towards morning: his skin hot and dry, and tongue coated with a white fur. ℞. *Potass. Hydriod.* gr. viij. *ex Mist. Camph. ter die.*

By the 20th of November the eruption was beginning to fade and desquamate. Soon afterwards more general improvement took place. On the 25th of December we are simply told he was presented.

Again, we must make this remark—that the case is totally valueless as evidence of the cure of syphilis by hydriodate of potass. Every body knows that mere salines and antiphlogistic remedies will remove lichen for the time. The hydriodate of potass may do the same thing. But that is not the cure of syphilis. These patients should be kept in hospital for some months, and watched. Then, and not till then, the experiment would be of worth. At present it must go for very little. We think it a duty to state these circumstances, and bring the true state of the case before our readers. Practitioners in the country, whose opportunities of observation are necessarily limited and desultory, are apt to be misled by the flattering accounts of the effects of many remedies that appear in the weekly Journals. We conceive we are called on to do our best to set the matter in its proper light, and to enable our readers to estimate these newly-introduced remedies at something like their real value.

---

### St. George's Hospital.

### Continued Fever.

The necessity of a profound acquaintance with the pathology of fever is soon felt by the physician or the general practitioner when engaged in practice. Cases of fever are those which frequently perplex him most, disappoint the expectations of his judgment, and baffle the resources of his art.

Independently of the vexata questio of the proximate cause of this disease, it is impossible to deny that modern anatomical researches have done a vast deal towards correcting our notions and guiding our treatment of fever.—Whether structural alteration does or does not constitute the initiative of fever, is practically a matter of little consequence. We now know that such alterations soon become developed, maintain the disease, and either retard the patient's recovery, or occasion his destruction. The opponents of morbid anatomy may believe, that the lesions of fever are comparatively unimportant, and may seek in their hocus-pocus doctrines the simple explanations that the scalpel gives. Let it be our's to ascertain by a comparison of symptoms and pathological appearances, the periods at which structural changes are developed, their characters and nature—let us ascertain this, and apply our knowledge in diagnosis and in treatment.

What an outcry was raised against those who looked for a local cause of fever. That they went too far, as fresh inquirers usually do, must be admitted. But they have succeeded in revolutionizing our notions and treatment. We have ceased to look upon typhus as a thing emblematic of debility and putridity, and so forth—we have tired of watching for critical days and critical demonstrations—we have ceased to cram people with bark and wine, because their tongues were brown, and themselves half comatose—those good old days and good old notions are for ever gone—and we look for alterations in the head, the chest, or the abdomen, to account for those symptoms which our forefathers, in their pathological ignorance, erected into substantive complaints.

We say that the physician, after the first few days of fever, begins to watch with painful anxiety for signs of structural changes in the head, the chest, or the abdomen. There can be little doubt that the latter are the more frequent and the more important. As a general rule, they are ulcerations of the mucous membrane of the intestines, originating usually in the glands of Peyer, and situated commonly at the lower portion of the ileum. For them the practical and well-informed physician watches, as the most frequent, and, perhaps, the most insidious of the organic lesions of fever.

The following cases selected to illustrate this point, are taken from a clinical report published by Dr. Aldis.*

Case 1. *Continued Fever—Abdominal Tenderness—Cure.*

Ruth L., æt. 29, single, Sussex, admitted, *May 22d,* 1833.—Pulse 120, small; skin hot, face flushed; tongue furred in centre, red at edges; urine free; catamenia obstructed; does not recollect when they ceased.

Complains of tenderness of the abdomen; no pain in the head; lips parched; great prostration of strength; much thirst; deafness; abdomen very hard.

Ailing a fortnight; illness commenced with shivering, followed by heat. Had variola six months ago.

The treatment was a dose of calomel followed by a black draught and by salines. The bowels were freely opened, and the motions were liquid and of a light yellow colour. On the 24th, the abdomen was tense and hard, and the face was flushed. The calomel and black draught were repeated, and the vin. ant. t. was added to the salines. On the 27th, the tongue was still loaded in the centre—bowels open—pulse 88, soft. On the 29th, the abdomen continuing hard, a dose of blue-pill and castor-oil were ordered. From this time she improved; cascarilla was given and borne—and, on the 10th of June, she was reported cured.

This case is as good an example as any of continued fever in a mild form. The abdominal tenderness and hardness shewed the disposition to affection of the mucous membrane of the bowels, which, in the next case, we find terminating in ulceration.

Case 2.—*Continued Fever—Hæmatirrhœa—Death—Ulcerations of the Bowels.*

Hannah B. æt. 24, admitted Feb. 2, 1832, in a state of heaviness and slight stupor, with some deafness, flushed countenance, and hot skin. Did not acknowledge any pain, except in the loins and legs. Was stated by her friends to have been confined to her bed for nine days, in much the same condition; but had complained of general illness, with occasional chills, for a week previously. Within a few days she had occasionally passed blood by stool. Has been bled, and had leeches applied to the temples: was ordered,

> *Hyd.* c̄ *Cretâ,* gr. vj. *Pulv. ipecac.* c. gr. iv. *sextis h. sum. superbibendo haustum Salinum. Olei ricini,* ℥ss. *cras.*

4th. Is lying on her back, with the legs extended, in a tranquil state; breathing accelerated and somewhat stridulous. Pulse 144, not hard; skin hot and dry; lips parched and glazed; tongue moist, but covered with brown fur. Is somewhat deaf, but quite conscious when spoken to, and answers questions correctly. Is stated to have been delirious and noisy during the last two nights. Heaviness in the head,

---

* An Introduction to Hospital Practice, &c. By C. J. B. Aldis, M.A. M.B. &c. &c. Octavo, pp. 125. London, 1835.

but no pain; pupils contracted; considerable pain on pressure of the abdomen, particularly in the left iliac region. Several bloody stools have been passed since admission; but she had one motion this morning, very offensive, without blood. There is some dulness on percussion throughout the left side of the thorax, with corresponding feebleness of respiration, which is quite puerile in the right lung. In the afternoon leeches and a blister were ordered to be applied to the abdomen. The dose of *Hyd.* c̄ *Cretâ* was increased.

*Detrahatur urina hac nocte nisi prius sponte defluat.*

Was very noisy during the night. Has had two motions, of dark colour, containing a quantity of dark blood. Respiration accelerated, and attended with the mucous râle, very abundant in both lungs, most so in the left. Face, lips, and hands purple; skin cold; pulse scarcely perceptible, fluttering.

*Sectio, 17 Hours post Mortem.—Head.* Considerable increased vascularity of the pia mater, without opacity of the arachnoid or effusion beneath it. No effusion, or if any, very slight, into the ventricles.

*Chest.* No adhesion between the right pleuræ; a very firm attachment of the posterior portion of the lower lobe of the left lung to the parietes. This portion of lung was found consolidated and infiltrated with black blood, several smaller spots of effusion being also found in the upper lobe. None in the right lung. The bronchial membrane of both lungs was extremely turgid, and of a reddish-brown colour, with a quantity of frothy mucus in the larger, and muco-puriform matter in the smaller ramifications. These appearances were more remarkable in the left lung. The heart rather soft, and left ventricle somewhat thinner than usual.

*Abdomen.* There were several large circular ulcerations about the ileum and upper part of the colon. The mesenteric glands were enlarged.

Dr. Aldis observes, in a note, that it is of consequence to attend to the condition of the bladder in cases of fever. Patients, particularly if oppression exists about the brain, are incapable of properly appreciating or mentioning the circumstance, and the bladder may become excessively, indeed injuriously distended. We have seen this occurrence ourselves, and its liability should be borne in mind by practitioners. The preceding case is a very good example of the lesions of fever. The patient dying on the 18th day, we find ulcerations of the mucous membrane of the ileum and colon, and irregular congestions in the lungs. The lesions of the lungs in fever are certainly not so important as those of the brain, and the lesions of the latter are of less consequence than those of the mucous membrane of the intestines.

Some cases of fever are related, in which pulmonary congestion was present. But there is not any thing about these cases requiring particular remark. In the next case there was observed a circumstance that now and then occurs.

Case 3.—*Continued Fever—Eruption of Furuncles—Death.*

James P., æt. 22, groom, Westminster, admitted July 17th, 1833.—Pulse 86, soft; skin warm and dry; countenance sallow; tongue slightly furred, red at the tip, moist; bowels much relaxed, hæmatirrhœa; urine free, clear.

Cough, slight expectoration; sweats at night over the head and chest. Dulness on percussion over the apex of each lung, but more so on the right; also at the lower lobe of the left under the axilla.

Was attacked two months ago with the Influenza, and expectorated blood, from which he was convalescent; on Sunday rode twenty miles, since which he has been attacked with more fever.

Has been bled and leeched.

℞. *Hydrarg.* c̄ *Cretâ*, gr. iij. *Pulv. ipecac. c.* gr. ij. *quartis horis. Emplast. canthar. pectori. Dieta lactea. Haust. sennæ cras mane.*

On the 18th, he was ordered calomel, black-draught, and leeches to the temples. He was, and continued to be, delirious. There was much abdominal tension and tenderness over the ileum.

On the 21st, the bowels were greatly relaxed, and an aromatic draught was given.

22nd. Bowels more quiet; pulse soft; was very delirious last night, but more composed this morning.

*Rep. Calomel. octavis horis tant. P.*

24th. Mouth not sore; was hot and feverish yesterday, when the aromatic mixture was ordered to be discontinued. P.

26th. Feels much more comfortable; pulse 80, full and soft; skin hot; tongue slightly furred; bowels open yesterday; abdomen hard; mouth sore.

*Garg. aluminis.—Sumat. Pil. calomel. o. n.*

℞. *Haust. sennæ cras.—Haust. salin.* c̄ *Vin. ant. tart.* ♏xx. *sextis horis.*

29th. An eruption of furuncles in various parts of his body; pulse frequent; tongue slightly furred; appetite improved.

℞. *Infus. rosæ, c.* ℥jss. *Quin. sulph.* gr. jss. *ter die.—Ext. coloc. c.* gr. x. *alt. noct.*

30th. Became very hot and feverish, and his bowels much relaxed, for which the quinine was discontinued, and he was ordered,

℞. *Haust. salin.* c̄ *Conf. aromat.* ℈j. *quartis horis.*

After this, a sloughing ulcer formed on the sacrum—he became more and more low, and on the 5th of August he died.

*Dissection.—Head.* The ventricles of the brain did not contain more fluid than natural. In the base of the cranium, there was a great quantity of bloody serum.

*Thorax.* The substance of the lungs appeared healthy; they did not, however, collapse when the cavity of the chest was opened. There was very extensive effusion of bloody serum in the left cavity of the chest. The right contained fluid, though considerably less in quantity. The inferior portion of the pleura of the left side was united by firm adhesions. The right auricle of the heart was filled with coagula, the left ventricle quite empty, and hypertrophic.

*Abdomen.* The liver was healthy, spleen rather soft, of a darker colour than usual, with two ulcerations on its surface. The small intestines were very much distended with air, and contained a quantity of fluid; they were of a very dark colour, and had sphacelated spots upon them. The lower three-fifths of the ileum were very much ulcerated; the edges of the ulcers not thickened; perforation of the intestines nearly completed in two or three places.

We must say that we doubt the safety of such purgatives as calomel and black draught in fever, after the earlier stages. We do not see on what principle they can be recommended. A patient has ulceration of the mucous membrane of the bowels. Is it likely that violent purgation, stimulating and disturbing an ulcerated surface, can be beneficial or even safe? God forbid, that were our own bowels ulcerated, we should swallow calomel and black draught. The milder forms of mercury, for example the hydrargyrus c̄. cretâ, combined with small doses of Dover's powder and rhubarb, and castor oil or aromatic aperients with henbane—such are the remedies which reasoning would recommend, and which practice and experience sanction. Purge a patient smartly at the commencement of continued fever, but after it has continued for some days, and especially if blood is mixed with the stools, beware of *purging* him further. Keep up the secretions, but do not *purge*, for drastic purgatives are decidedly dangerous.

Case 4.—*Chronic Peritonitis from Perforation of the Intestines.*

Elizabeth D., æt. 28, single, admitted, *Feb. 20th,* 1833.—Complains of diarrhœa, accompanied with pain in the bowels after every motion: the abdomen is very painful to the touch, sometimes feels hard; has a slight dry cough; is very much emaciated; catamenia obstructed five months; tongue moist; urine clear but scanty. Last July had an attack of bilious vomiting, since which time she has become much thinner. In November last the diarrhœa came on, which has continued to the present time with very little improvement.

March 14th.—Died 4 o'clock A. M.

*Sectio.*—*Chest.*—The lungs were found to be perfectly healthy; rather more fluid than usual in the pleural cavity; the heart was very small, but apparently healthy.

*Abdomen.*—The liver was of its natural size, very pale. The peritoneum was very much thickened, universally adherent, and in some parts tuberculated. The large and small intestines were firmly glued together by very strong adhesions. An extensive abscess was found within the cavity of the peritoneum, which communicated by several large ulcerated openings, surrounded by hard thickened edges, with the ilium; much ulceration round the ileo-cæcal valve, and also at the sigmoid flexure of the colon. The peritoneal coat of the small intestines, as well as the mesentery, was studded with Baron's tubercular accretions. The mesenteric glands were much enlarged by a scrofulous deposit.

It is by no menns certain this was really a sequela of continued fever. It is not evident to our mind, that the disease may not have originated in the peritoneum.

Perforation of the intestine is well known to occur sometimes in fever. The ulcerations of the mucous coat involve the muscular, and at last peritoneal. The patient suddenly complains of most intense abdominal pain—extreme collapse rapidly takes place—and death speedily occurs with all the symptoms of violent peritonitis. The contents of the bowels are found to have escaped into the peritoneal cavity, and to have given rise to the inflammation of the membrane. Such are the usual characters of these cases. With the following extract from Dr. Aldis's work, we conclude the present report. The author presents a resumé of the pathological changes that the organs undergo in fever.

"*Head.*—Increased vascularity of the membranes of the brain, with serous or purulent effusions. Opacity and thickening of the arachnoid. Sometimes minute red spots are found in the substance of the brain, when vessels are cut through, distended with blood. Sometimes the veins connected with the brain are very turgid. Fluid in the ventricles. Effusions of coagulated lymph.

*Chest.*—The lungs may be full of blood, or frothy serum. In a subsequent period they become condensed from a deposition of coagulable lymph. The trachea and bronchi may exhibit patches of ulceration. These pulmonary obstructions will account for the lividity of the countenance, noticed in some cases of fever.

*Abdomen.*—Congestion and ulceration of the intestines. The progress of the ulceration has been previously described.

The following is a tabular view of the result obtained by M. Andral, from an examination of seventy-one cases, given in his work on Fevers:—

| | |
|---|---|
| The stomach presented ulcerations in .......... | 10 cases. |
| Duodenum ............ | 1 |
| Jejunum ............... | 9 |
| Ileum (its lower part) .... | 38 |
| Cæcum ................. | 15 |
| Colon, Ascending ........ | 4 |
| ——, Transverse........ | 11 |
| ——, Descending ..... | 3 |
| Rectum ................ | 1. |

It is a question, whether this appearance of the mucous membrane of the intestines precedes, or is a consequence, of fever.

*Symptoms of Perforation of the Intestines.*—If the patient is able to describe his symptoms, he complains of sudden and very acute pain in the abdomen, succeeded by vomiting. The pain continues with great severity in consequence of partial and then general peritonitis supervening. The pulse is small.

The *Mesenteric Glands* are found enlarged in consequence of the absorption of the irritating discharge from the ulcers. The absorbents proceeding from the ulcers to the glands are sometimes filled with matter.

I have never seen any remarkable alteration in the *Liver*. The *Spleen* is occasionally very much enlarged, or softened, having a pulpy appearance. It is sometimes very hard.

The *Blood* may be very dark and fluid in the arterial trunks."

### Pennsylvania Hospital.

Surgical Report. By Dr. Kirkbride.

The report from which we shall extract or notice the succeeding cases is contained in our valued Transatlantic contemporary, the American Journal of the Medical Sciences for May of the present year.

### Cases of Dislocation and Fracture.

Case 1. *Dislocation of the Head of the Os Femoris downwards and backwards.*

James C., æt. 35, a very muscular labourer, admitted at noon of the 23d of January, 1835. One hour previously a roof fell in upon him. He thought that his right thigh was at first separated from the other, and that then his whole body was pressed flatly to the earth.

On admission, the dislocated thigh was flexed upon the pelvis, and rested upon the uninjured one; the leg was flexed upon the thigh; the knee was below its fellow; the toes turned inwards; the back of the foot, over the origin of the metatarsal bones of the injured side, rested upon the anterior part of the hollow of the tarsus of the opposite limb; the limb could not be extended, and the attempts to effect it produced excruciating pain; the point over the acetabulum was more depressed than natural; the head of the bone was thrown downwards and backwards, and with the trochanter major could be felt with perfect distinctness moving under the fingers when attempts were made at rotation. The head of the bone appeared to rest upon the posterior part of the body of the ischium, between its tuberosity and its spine. The distance between the trochanter major and the anterior superior spinous process of the ileum, was one and a half inches more than on the sound side. The muscles about the joint, and those of the thigh, were thrown into spasmodic action by all attempts at motion. The patient was unable to lie upon his back, always choosing the sound side, with the injured knee resting on the bed. He was bled to 32 ounces, and put on the solution of the emetic tartar. At half-past eleven, a. m. of the next day, he was placed upon the table, and the pullies applied in the ordinary manner; great difficulty however was experienced in securing the extending band above the knee, owing to the shape of the thigh, and the large body of muscles at the part. Some extension was made however, and at the same time it was attempted to lift the head of the bone from its position, by raising the whole thigh. A second attempt at extension was now made, but the bands slipping, as they did before, they were removed entirely, with the intention of making the attachment at the ankle; at this juncture it was observed that the form of the hip had changed; the toes were turned outwards; the limb could be extended, and rotation performed; in fact, it was discovered that the reduction had been effected. Neither the patient nor attendants were conscious of the moment when the reduction took place; no sound was heard, nor any particular motion observed. The patient suffered scarce any pain during the operation. At first there ensued loss of sensibility and motion in the limb, but it gradually recovered both.

Dr. Kirkbride, in his observations, recapitulates thus the essential symptoms of this rare, and hitherto undescribed dislocation. Those symptoms are as follow:—the position of the thigh across the sound one, the flexion of the leg upon the thigh, the lengthening of the limb, which could not have been less than one inch, proved by the position of the knee below its fellow, and by that of the foot of the injured side under the sound one, the increased distance between the trochanter major and anterior superior spinous process of the ileum, (one and a half inches,) the difficulty of making rotation, the impossibility of producing extension, and the facility with which both the head and trochanter could be felt, particularly the last of these facts, are points sufficient to indicate with precision the locality of the head of the bone, as being upon the posterior part of the body of the ischium, between its tuberosity and spine, and distinguishing it from all other dislocations of the joint. The paralysis too, (which was probably owing to the unnatural situation into which the sciatic nerve was thrown,) was more complete than is usual, and of longer continuance.

Dr. Kirkbride refers to two analogous cases—one published by Mr. Keate, senior surgeon to St. George's Hospital, the other by Professor Warren of Boston. Mr. Keate's case is very interesting, and as it is short, we shall insert it here in connexion with the case before us.

"I was called," says Mr. Keate, "into the country, on the 13th of February, to see a gentleman who had met with a severe accident, by his horse having fallen backwards with him, and upon him, into a deep and narrow ditch; where he had remained, as he supposes, for near a quarter of an hour before he was discovered, when he was nearly exhausted by pain and by fruitless exertions in calling aloud for aid. The horse was lying on its back upon him, with his heels struggling in the air; but the gentleman, who is strong and muscular, appears to have retained a firm hold of the bridle, and thus to have kept down the horse's head, and restrained, in some degree, the violent efforts of the animal.

He had been brought home, and was on his bed when I arrived. On examining the limb, I found it unusually elongated, at least from three to three inches and a half. The thigh was much flexed upon the pelvis; the leg as much bent on the thigh. The whole limb was carried outward, or apart from the other, more than I had ever observed in any case of luxation. The knee and the foot were much everted, the trochanter extremely sunk, the soft parts being elevated in a circle around it. I found that the head of the femur was displaced in a very unusual manner, to a situation inferior to the ischiatic notch; and I felt it lying close to, and on a level with, the tuberosity of the ischium, where it was capable of being freely moved under my fingers.

Without noticing the usual preparations for reducing a luxation, it will be sufficient to say, that, in the first attempt, the head of the bone was thrown into the foramen ovale. A second extension enabled me to place it *nearly* in its proper position in the acetabulum, but it could not be perfectly replaced; and on gently moving it, and placing my ear on the trochanter, I felt and heard a distinct grating, as if of ruptured cartilage. By drawing the upper part of the femur outwards, (by means of a round towel thrown over my neck,) and pressing the knee sharply inwards, the head of the bone was replaced, with a snap, in the acetabulum; but even after this I was able to elongate or pull down the limb, and it was evident to me that this was owing to a portion of the cartilaginous labrum having been broken off during the violence of the accident.

The gentleman was quite aware, and mentioned, after the first step, as I may call it, of the reduction into the foramen ovale, that the head of the bone was not properly replaced; and he stated that the luxation had taken place by the same route, first into the thyroid foramen, and afterwards, while struggling in the ditch, from thence downwards to the situation in which I found it. The case has proved very favourable, but there was a severe injury at the same time to the knee, which threatens still to be troublesome."*

There are obviously some distinctive characters between these cases. In Mr. Keate's, the head of the femur was not situated in the lesser sciatic foramen, but in some indeterminate situation contiguous to the sciatic tuberosity. Still it must be owned that some similarity exists between the cases. The other instance referred to by Dr. Kirkbride is related by Professor Warren, of Boston. It was one of great difficulty and some obscurity, and in which the attempt at reduction was not successful. From the account given of it, it seems to have somewhat resembled that recorded by Mr. Keate. The great increase in the length of the limb, (three inches,) and the position by the

* Med. Gaz. Vol. x. p. 19.

foot, prove the head of the bone to have occupied a situation materially different from that described by Dr. Kirkbride.

The next case reported is a common one—dislocation of the head of the femur on the dorsum of the ilium. We may pass it by.

Case 2. *Odd Kind of Union of a Fractured Patella.*

In a patient who was received for other things, the following particulars were remarked in reference to the patella of the right knee. It had been formerly broken, and was partially united thus:—

The fracture had been transverse, and nearly in the middle of the bone; the two portions at the time of observation were connected together by a firm and nearly round ligamentous cord, about the ordinary size of the ring-finger, and extending between the internal angles of the fragments; in all the rest of their extent no connexion whatever could be detected. The consequence of this is, that the external angles diverge widely from each other, being three and three-fourths inches apart when the leg is flexed, while the internal are only two and a half, and when the leg is extended, the former are two inches asunder, and the latter one and a half only. The fragments may almost be brought into contact by firm pressure. Notwithstanding this state of the parts, he is able to walk as rapidly as ever, and without perceptible limping. He suffers but little inconvenience from it, although he thinks it rather weaker than the other; and on one or two occasions, when carrying heavy weights, it has given way under him, and required rest for a few days afterwards; in ascending stairs he generally puts the left foot foremost, and brings the other up to it, although this is not absolutely necessary. The history he gives of the injury is, that having fractured the patella by a fall, the limb was kept in splints for four weeks, when, contrary to the advice of his attendant, he removed them, and began to move the joint. Although he suffered some pain at first, he never afterwards consulted his physician, and in three months from the occurrence of the accident he engaged in his usual occupations.

Case 3.—*Fracture of the External Condyle of the Femur—Moveable Fracture-box.*

A case of fracture of the external condyle of the femur is reported. It exhibits no feature of importance, and we merely mention it, to make it a peg for the following remarks of the reporter on the use of moveable fracture-boxes. We should observe that in the case in question the extended position and the long splint of Dessault were the means at first employed, but that when union appeared to have taken place, the motions were both limited and painful. To remedy this a box was contrived, the angle of which (at the knee we presume) admitted of alteration. Motion was thus gradually and safely obtained.

"The stiffening," says Dr. K., "after fractures is of so frequent occurrence, and so annoying to both patient and surgeon, that any means likely to lessen its frequency or to restore the action of the part are very important. The moveable fracture-box employed in the above case is often exceedingly useful, and may be applied as soon as the callus has become tolerably firm, previous to which the extended position will almost always be found to be the safest. From the result of two or three cases of severe compound fracture of the leg, in which the splint of Professor Smith, of Baltimore, was used, I am inclined to believe that it will be found exceedingly useful in obviating the inconvenience above noted; as in two of the cases there was no rigidity or pain in the joints, although their confinement had been protracted. This splint is also worthy of commendation, from its very great convenience in compound fractures, particularly of the leg, (in which alone I have as yet employed it,) from the facility with which the parts may be inspected, the soiled dressings removed, and thorough ablutions of the part performed without in the slightest degree incommoding the patient or disturbing the coaptation of the fragments. The position too is generally agreeable to the patient, and motion of the joints may easily be made, whenever deemed adviseable."

Case 4. *Fracture of the Humerus from mere Muscular Exertion.*

E. M., æt. 21, admitted January 14th, 1835. He is tolerably robust, and has always enjoyed good health, except that about four months previous to his admission, he had an attack of rheumatism. He had never before had any fracture excepting of the skull, from a blow by a brick-bat when about five years old, when several portions of bone were removed; these parts are now firm. On the morning of his admission, he was throwing oyster-shells from the wharf on the ice in the Delaware, and, in one of his efforts, which at the time he did not think violent, by simple muscular exertion he produced a fracture of the os humeri, about one-third of its length from the lower extremity. He came to the hospital immediately afterwards, nothing peculiar occurred during the treatment, and the bone became perfectly firm in about the usual period.

### Injuries of the Head.

Case 1. *Wounds of the Scalp—Symptoms of Phrenitis.*

A coloured seaman, æt. 26, was knocked down by a cart on the 1st of November, 1834. The scalp was wounded, and a portion of the right parietal bone, denuded of periosteum, exposed. He was received into the hospital half an hour afterwards, insensible and apparently intoxicated. He gradually recovered his sensibility, and no unpleasant symptoms were observed till the evening of the 6th, when he had violent pain in the head, and frequent vomiting; the bowels were not open—the scalp-wound was suppurating. A stimulating enema, the mustard pediluvium, and cupping on the head, were the means employed. At 10, p. m. the symptoms were aggravated—pulse 80, with some strength—surface warm—some twitches in the left arm—restlessness and irritability.

*V. S. ad* ℥xxxv.—*C. c. capiti raso ad* ℥x.—*Postea lot. frig.*—*Mist. efferves.*

The pulse rose during the venesection, which, however, was continued until a decided impression was made on it. The pain was much relieved, but still it did not disappear, and next morning it was described as deeply-seated in the eye-balls, and aggravated by the light—pupil a little contracted, and the conjunctiva injected—pulse 80, slightly irregular—tongue furred and whitish—skin warm.

*Mercurial purge—Solut. ant. tart.*

After this he steadily improved—the scalp-wound healed—and on the 13th January, he was dismissed cured.

We notice this case, not on account of its containing any thing extraordinary or striking, but because it will enable us to make two practical remarks. In the first place, the reporter of the case does not appear to be fully aware of the value of calomel in the treatment of the inflammation of the brain or of its membranes that may follow an injury of the head. Mercury is extremely useful in these cases. Conjoined with bleeding it accelerates the cure, and it also diminishes the quantity of blood which it is necessary to abstract—in other words, it saves both time and blood. There are some cases indeed where simple antiphlogistic treatment will not of itself be sufficient to check the inflammatory action. Mercury is very serviceable, and every surgeon ought to be well aware of that.

The second remark we have to offer is not peculiarly applicable to this case. But we would observe that two or three days after a scalp-wound, symptoms are apt to make their appearance, which the surgeon may and frequently does mistake for phrenitis. There are nausea—fever—pain in the head. These symptoms usher in erysipelas. Phrenitis usually occurs at rather a later period. But of course there is great uncertainty about this, and the surgeon should be upon his guard. It is better to sin on the side of considering the disease phrenitis, and treating it actively in consequence, than on that of holding it too cheap, and allowing, perhaps, irretrievable mischief to occur.

Case 2. *Fracture with Depression—Death on the 30th day.*

A labourer, æt. 31, fell from a height of about thirty feet, was insensible for a short time after the accident, and vomited blood, which probably proceeded from the nose, as that was found to be bleeding afterwards. In the course of the day he was taken to the hospital. He was then sensible, and answered questions correctly.

In the evening there was slight reaction, and he was ordered to be cupped, &c. He went on pretty well till the 5th day, when there were restlessness, slight delirium, pyrexia, and pupils a little contracted. *Bleeding, purging, &c.* On the 7th day, the symptoms were aggravated—sleeplessness—slight subsultus—picking at the bed-clothes—head hot—pulse 100—tongue dry—sordes on the teeth. *Cupping, &c. followed by blisters.* The symptoms were slightly and partially relieved, but on the 20th day we find him with low fever—disposition to get out of bed—low muttering delirium. We see no necessity for pursuing the details of the case. The symptoms of low fever continued—he sank progressively—and on the 30th day after the accident he died, having exhibited strabismus for the two days previously.

*Dissection.* Passing over a multitude of unimportant particulars, we shall content ourselves with stating what alone is essential—the condition of the head.

"Upon removing the scalp an effusion of blood was found immediately over the anterior part of the frontal bone; upon removing the pericranium, which was not detached, a fracture was observed, with very slight depression; this fracture, as discovered at a later stage of the dissection, commenced on the right side, two inches above the orbit, near the external part of the os frontis, and extended obliquely down to the orbit; from this another fracture extended so as to meet the orbit one inch nearer the external canthus. The first, after reaching the orbit, passed in a straight line through the whole body of the sphenoid bone, one-fourth of an inch from its centre. On the left side of the head was another fracture, a little more external, but taking the same general direction, and terminating at the body of the sphenoid. The depression of the frontal bone was about the one-sixteenth of an inch.

*Brain.*—Vessels on the dura mater and longitudinal sinus empty; the lateral sinuses contained coagulated blood; the arachnoid on the surface smooth, very moist; pia mater paler than usual; cortical substance of good consistence and natural colour; medullary pale, firm, very few dots; brain throughout moist. The arachnoid covering the anterior lobes of the brain, corresponding with the fracture, and surrounding the optic nerves, was dry, granulated, and adherent. The central parts were pale and of perfect consistence. *Cerebellum.* On the inferior surface of each lobe was a coagulum of blood, superficial, nearly half an inch in diameter, immediately around which was doubtful softening. The ventricles contained ℥ij. of serum, and about the same quantity existed at the base of the brain."

There are two rather singular features in this case. First, although there was fracture with depression, and although there was extravasation of blood into the lateral sinuses, as well as on the cerebellum, the patient recovered his sensibility, and at first had the usual symptoms of concussion rather than those of compression. This we say is not common, but instances of the sort are seen now and then. The second peculiarity is this. The patient died with low fever, and those symptoms which mark ramollissement of the brain, or at all events which mark the changes in that organ or its membranes occasioned by slow inflammatory action. Yet the account of the dissection, if correct, shews that only a dry, granulated, and adherent state of one portion of the arachnoid, and dubious softening of the surface of the cerebellum were the only lesions. We should certainly have anticipated the existence of more.

To the practical and philosophic surgeon the interest connected with the study of the injuries of the head is unlimited. Each fresh fact offers something new, and from each the most observant and experienced surgeon gathers information.

### CLINICAL LECTURES.

### 1. MR. TRAVERS ON CLINICAL INSTRUCTION.

Mr. Travers at the commencement of the present medical session, delivered to the students of St. Thomas's Hospital a clinical lecture on clinical tuition. It appears that, from some cause which it is not our business to inquire into, the school of that hospital has somewhat declined. There is evidently on the part of students a migration to the West. The Borough schools, formerly so flourishing, would seem to be losing a portion of the pupils, and this important *pabulum* of lecturers and lectures is more rife at the other end of the metropolis.

Small states, as well as large, submit to the revolutions of time and taste. We have dynasties overthrown in a parish, as in a nation, and the victory of the Browns over the Thompsons, is just as much a matter of science and of strength, as the conquest of one people by another. It appears from the weekly journals that in this metropolis a fearful rivalry exists between the hospital and the private schools. Far be it from us to enter the lists in favour of either, but we may make a few observations on their present and their past position.

It is not many years since interest was much more potent than it now is. The Colleges peacefully issued their decrees, which were little talked of and less questioned. Students were to be educated at a few specified places, and whether the teachers were good or bad was a matter of comparatively little consequence. One school, perhaps, would have more reputation than another, but the establishments were few, and the differences between them were, after all, unimportant. The mode of procuring bodies for dissection reduced the anatomical teachers pretty nearly to a level, for the resurrection-men had this great merit, that they served with equal diligence all who paid them. If a private teacher fee'd these ruffians well, he was certain of a good supply of bodies, and he consequently had the means of teaching anatomy quite as well as his hospital rivals.

But "a change came o'er the spirit of the dream." The genius of reform walked forth, and he laid his hand upon the Colleges. The private teachers had been formerly licensed with some degree of difficulty and reluctance. Monopoly or the semblance of it, was no longer possible, and, properly enough, the private teachers were allowed to take their course, and increase and multiply as circumstances should direct them. Many of our readers must recollect the year on which this license was first felt and carried to its full extent. The teachers overspread London like locusts, and the number of the new private schools was astounding. Gentlemen appeared to think that all they had to do was to lecture—classes *must* come.

A few years have elapsed. The private schools have dwindled and are dwindling, and it is not difficult to perceive that in a very few years more there will be five or six large schools in London, and that many which are now struggling for existence, will inevitably sink. The experiment has been made, or rather it is being made at this moment, whether the means of private teachers will enable them to compete with public teachers. It is only a fraction of that great trial which is going on throughout the country—it is a part of the great struggle between capital and no capital. The case of the small and the great manufacturers is perfectly analogous. That case has been very ably stated in successive numbers of the two political and critical reviews, the Edinburgh and the Quarterly. However we may deplore, and all men of humanity must deplore, the hardships experienced by the small manufacturers, there is not a doubt that they are falling fast, and the great Cotton Lords must win the day. The accumulation of capital is the natural tendency of things, and capital is power.

The teachers connected with the great hospitals possess numerous advantages over their private competitors. In the first place, there is the advantage of concentration. Systematic lectures

—clinical lectures—hospital practice—dissections in common and in morbid anatomy, all come together, for mutual aid. The student does not lose his time. In the next place, the hospital affords its teachers opportunities for illustration of every description. To the lecturers on physic and surgery, living patients, the alterations of organs in the dead-house, and the preparations of those changes in the museum. The anatomical lecture-table is supplied with viscera and with all the matériel for demonstration, independently of the regular supply of bodies, which he shares, probably equally, with his neighbours. When we add to these advantages, the actual monetary means at the disposal of the combined lecturers of large establishments, we shall understand what *their* capital is, and how heavily it must weigh in the scale, against the almost unassisted industry of the private lecturer. Indeed it may be said that the contest between the hospital and private schools is decided.

But a graver contest is impending—one between the great public schools themselves. It will be curious to watch this battle. The schools which are to take the lead in London *must* have men of talent connected with them. The mass of the profession are very well acquainted now with what a teacher should be, and are disposed to look with very jealous eyes at any thing approaching to the appointment of fainéants. Public opinion is stronger every day in the profession. The contest between the metropolitan schools will make *some* of those schools appeal to public opinion, and woe to the establishment against which that opinion is directed. The qualifications for a popular lecturer of these days, are widely different from what they were, even a few years back. Whatever his sterling merits may be, there must be some ad captandum talents too. This is felt at the hospitals and schools, and medical officers and lecturers are daily becoming more cautious whom they admit into their ranks. The false choice of one is the gain of another, and the appointments of mere interest may be said to have nearly passed away. Self-preservation forbids them.

We have wandered, however, from the immediate subject—the introductory lecture of Mr. Travers to the clinical course. We extract the following sentiment of that gentleman on the value of clinical instruction.

"With regard to CLINICAL INSTRUCTION, it may fairly be stated, that no mode that can be devised is more helpful to the student in acquiring a thorough knowledge of medicine,—the term *medicine* including both medicine and surgery. The present era has been, and will continue to be, distinguished in all times for the great contributions made to this department of science. Its advantages are most obvious. The mind is far more difficult to be fastened on a subject when it is addressed only through the ear (as in the general systematic lectures), but what obtains the cognizance of other senses (as in clinical instruction) is calculated never to quit the mind."

People are too apt to swim with the stream. A little while ago no clinical lectures were delivered—now every body wishes to give clinical lectures, and these are the veritable Amphitryon, the high road to knowledge. There is a great deal of exaggeration in the present mania for clinical lectures. As we were amongst the most earnest in recommending the adoption of the clinical system, we shall not be accused of an overweening fondness for things as they were, in questioning whether that system may not be pressed too far—whether the hobby may not possibly be ridden too hard.

Clinical lectures are lectures on particulars, in opposition to systematic lectures, which are rather devoted to general principles. It is true that the whole is made up of parts, and that instruction upon those parts must greatly contribute to a comprehension of the whole. But it would be absurd in theory to make the study of the part superior to that of the whole, and practically, the absurdity is even greater than it is in theory. We have seen something of the effects of clinical lectures, and valuable as they are, we are convinced that they cannot be made a substitute for general lectures or for individual study. We greatly doubt whether formal clinical lectures have not already been carried too far. Pupils appear to us to be rather sick of them, and if ten clinical lectures are delivered weekly in any one hospital, we venture to say that not more than two or three are generally well attended. If a man is very popular as a lecturer, and if his practical knowledge is great, his clinical benches will be filled. But under ordinary circumstances, clinical lectures are not well attended.

Mr. Travers alludes to the old maxim that "seeing is believing:"—

> Segnius irritant animos demissa per aures,
> Quam quæ sunt oculis subjecta fidelibus—

This is less applicable to clinical lectures, strictly speaking, than to clinical instruction. It appears to us that there is not quite enough of the latter, and a tendency perhaps to accumulate upon the student rather too much of the former. At some of the hospitals the diurnal visit is a gallopade. The surgeon and the students walk the wards, or rather run them, as if the cry was "sauve qui peut." A few hurried questions are asked, and scarcely answered, a few hurried things are done, the pupils scamper, jostle, and talk, and a certain amount of space having been traversed by these peripatetic philosophers, what is called the "visit" of the day is over, and like most morning visits, it ends by leaving those who receive or those who pay it very little the wiser. This is the portion of hospital practice which we think might be much improved. A well-regulated system of bed-side instruction might be made more profitable, and infinitely more attractive than the present indulgence in formal lectures. We do not wish to be misunderstood. We are not advocating the discontinuance of clinical lectures—far from it; but we think that they are carried rather too far, and that their frequency is defeating the object for which they were instituted, by fatiguing and disgusting the already too be-lectured student. One thing is quite certain, that a great many clinical lectures and lecturers are very badly attended. With these observations into which we have been almost unconsciously betrayed, we proceed to notice some portions of clinical lectures delivered by various gentlemen, and published in the contemporary journals.

### II. CLINICAL LECTURES OF SIR B. BRODIE.

### 1. *On Epulis.**

This disease, according to Sir B. C. Brodie's experience, occurs usually in women; but he has seen it in men. The following is the history of the complaint presented us by this excellent practical surgeon.

"When you look at the disease in its early stage, it appears as if a part of the gum were more prominent than the rest; the prominent part is covered by a membrane like the gum, and when cut into, it is not very different in appearance from the consistence and structure of the gum itself; at least, so far as the eye can discern. On these accounts, as it looks like the gum on the surface, as it cuts like the gum, and as it is connected with the gum, so it is supposed generally to have its origin in the gum, and I cannot say that that is not its source in some instances; but yet I must own that in cases which I have had an opportunity of examining, it has appeared to me that the disease originated in the alveolar processes. I have observed that the disease is always situated by the side of one tooth, and generally a tooth is pushed out of the socket as the disease advances. The history of the present patient tends to confirm this opinion, for she says that the teeth became loose and dropped out, and then up grew the tumour. Here is a specimen (*presenting it*) of the disease, attached to the jaw, and in which it evidently appeared to have originated in the alveolar process. In the operation, I removed with the tumour the portion of jaw belonging to it; and I found in that part of the jaw which was sawn through, and where it was not supposed that any tumour existed, that there was a substance exactly like

* Lancet, Nov. 21, 1835.

the tumour itself, in the alveolar process. I sawed through the bone where I thought it was healthy, and there I found the socket of an alveolus, in which the structure was so exactly like that of the large tumour, that I thought it right to destroy this part. The tumour is at first small; as it advances the teeth drop out, and the tumour at last extends from one side of the jaw to the other. If the disease go on it will ulcerate and increase in size, and although in this woman it remained stationary, as any tumour may, yet it may go on to attain any magnitude. I have seen a tumour occupy the entire half of the lower jaw, so that the patient could not close the mouth; and it may go on further still, and run the course of any malignant tumour. Consider what any malignant tumour of the jaw will do, and that is what this tumour also may do. It may occupy the cavity of the mouth itself, press upon the cheek, and ultimately produce destruction of the face, so that you have a large ill-conditioned ulcer, with a tumour at the bottom of it. In short, it may run the course of any malignant disease."

Sir B. Brodie proceeds to state that although the tumor, if left to itself, will ulcerate, slough, and produce destruction of contiguous parts, just as scirrhus or fungus hæmatodes, yet it is only so far malignant; for it will not contaminate the constitution like those truly malignant disorders. If a scirrhous tumor is removed, the probability is, that another scirrhous tumor will appear in the cicatrix, or in some other organ or part. But epulis is one of those half-malignant diseases, which, although not capable of a natural cure, and ending in the destruction of the textures with which they are immediately involved, do not seem to produce that condition of system which gives rise to the formation of similar tumors elsewhere. This is a very consolatory fact, and exerts, of course, an important influence on our views of treatment.

If tumors then like epulis are removed, they do not reappear. But they must be removed *entirely*. When the tumor is taken away at a late stage, the disease does occasionally return. The reason is, not that the disease is then more malignant, but the eradication by the operation has not been quite *complete*. The same thing occurs with the tubercles which form on the cheeks of old persons. Remove them early and remove them perfectly, and they do not grow again. If removed late, they may not then be removed so perfectly, and so under these circumstances they do grow again. The following is Sir B. Brodie's method of operating in the instance of epulis.

"The mode of proceeding must depend on circumstances,—on the size of the tumour,—on its extent,—and, partly, on its position. In the greater number of cases of this kind that I see,—in private practice at least,—the disease is in the early stage, and, of course, then, the tumour is of small size, and may be easily destroyed in the following manner:—First of all, all the teeth which seem in any way to interfere with the disease must be drawn. This step must be taken before you can do any thing else. You thus expose the tumour completely, and then, in ordinary cases, you may proceed in the following manner. If the teeth, where the tumour is formed, have been dropped out for some time, the alveolar processes have become absorbed; otherwise, of course they remain, and this circumstance you must bear in mind. I am now speaking of cases where the tumour is small. I place the patient before the light, and then cut off the excrescence, so far as I can get at it. If the alveolar processes remain, of course I use such a knife as can be carried to the bottom of them; but if they have been absorbed, a straight common knife will do. You then wait for the bleeding to subside, and if there be a great deal of hemorrhage, you may postpone the next step of the operation to another day, when it may be done quite as well as on the first occasion. The next step of the operation is to apply the caustic potass to the surface of the bone from which the tumor arose. You may apply the actual cautery, or nitric acid; but I prefer the caustic potass, which answers the purpose fully as well as the actual cautery, and frightens the patient much less. I think, moreover, that you know more exactly how far you go with the caustic potass than you do with the actual cautery; and be assured, also, for I speak from having employed it many times, that it answers the purpose perfectly. You should have a piece of caustic potass, with a point, so as to enter the alveolar process. It should be cut in the shape of a pencil, and be a piece of a tolerable length, and it should be fixed, at right angles, in the end of a pair of dressing forceps. Do not trust to your hands to hold it tight in the forceps, but let it be fastened on by a ligature, passed round several times; apply it to the surface from which the tumour had been removed; but if the alveolar process remains take care that the caustic penetrates to the bottom of the process. Many prefer the caustic potass to anything else, because it does not coagulate the blood, and does not prevent the caustic from acting, and because also, it will penetrate somewhat into the substance of the parts; whereas the nitric acid coagulates every substance with which it comes in contact, and does not sink into it; it is more limited in its effects. You may conceive that the caustic potass is very likely to run about, to surround the cheek, to burn the tongue, and to injure parts beyond those which it is your intention to injure. It will dissolve in saliva, in the blood, and in the urine, and if it were to run about it would produce very great evil. How, then, are you to obviate the effects of such an accident? Why, just as you always would avoid the effects of its application where you do not want it to operate. Whenever you employ it for the destruction of living parts, have something at hand that will act as an antidote to it, and stop its operation. If you use caustic potass, you need only have some vinegar within reach, with bits of lint to dip into the vinegar and apply to the neighbouring texture. If you employ nitric acid, have some carbonate of potass, or chalk and water, ready to apply to protect neighbouring parts. You should never use caustic without having something by you that will destroy its properties, when the caustic is in danger of interfering with the neighbouring textures. There are some cases in which you apply the nitrate of silver (which is not a powerful caustic, and not much used for the destruction of parts) to the inside of the eyelids. Always have something at hand on such occasions to stop its operation, and the best antidote with which I am acquainted, is common oil, which stops its action presently. But, to return.

Having removed the part with the knife, apply the potass to the surface, by which you will make a slough of the neighbouring parts, and destroy the surface of the bones. If the disease has descended to the alveolus, and the alveolar process is not absorbed, a narrow piece of caustic is to be introduced into the bottom of the process. This may be done at the time of excising the tumour, if there be not much hemorrhage; but if there be, then it is better to defer the application to another day: no harm arises from waiting, and you never can apply the caustic to much advantage when there is much hemorrhage; that was the reason why I only applied the caustic slightly to-day. You should always examine the part afterwards, in order to ascertain if you have left any portion of the tumour undestroyed. If you have, it may be removed by a knife, or by the caustic potass, afterwards. It is not often necessary, but still, where the comfort or the life of the patient is at stake, you should exercise this precaution."

In cases where bone enters into the composition of the base of the tumor, Sir B. Brodie employs a pair of bone-nippers, a modification of the common stump-bone-nippers, but constructed with a double lever by Mr. Weiss. The double lever gives great additional power to the operator. Sir B. Brodie particularly cautions the operator not to leave any disease in the jaw. It may be necessary to remove very large portions, or the whole of it.

### II. Sir B. Brodie on Affections of the Rectum.*

### *Spasmodic Contractions of the Sphincter Ani.*

Every surgeon knows what a painful and troublesome complaint this is. According to Sir B. Brodie's experience it is most frequent in women, especially in those disposed to hysteria. Frequently a small ulcer of the mucous membrane of the rectum is found to exist in connexion with this affection of the sphincter. The ulcer is situated opposite the point of the os coccygis. It is exquisitely painful on pressure, the passage of the fæces, &c. The following is Sir B. Brodie's treatment of the complaint.

"When the patient does not suffer excessively from this disease, you may sometimes relieve her in the following manner:—Give her purgative medicine, so that she may never have hard or figured evacuations, and let an opiate suppository be introduced at night. I have formerly used a suppository with extract of belladonna, with manifest advantage; but I own that I am not in the habit of frequently employing this remedy. Even used in the form of a suppository, the belladonna sometimes produces very serious symptoms, by its influence on the brain. In addition to what I have mentioned, the patient may introduce a bougie into the anus, to dilate the orifice of the bowel, each time before she goes to the water-closet.

These remedies, however, are of no avail in bad cases of this disease, and then it is absolutely necessary to resort to some more certain means of cure. It may always be relieved by a simple operation—the division of the sphincter ani muscle. You introduce a straight probe-pointed bistoury into the anus, and cut through the fibres of the muscle, taking care not to penetrate beyond them. The fibres are of considerable thickness, and you cannot cut them through at one incision, nor should you attempt it; the knife must be drawn across the muscle two or three times before the operation is completed. It is generally sufficient if you divide the muscle on one side. It is better to divide it laterally than either in the posterior or anterior direction. The wound does not readily heal if the division be made towards either the perineum or the os coccygis; nay, more than that, if in the female you divide the muscle towards the perineum, and consequently towards the vagina, you make the patient miserable for life, for there is incontinence of fæces ever afterwards; whereas, if you divide it in any other direction, this inconvenience is altogether avoided after the wound is healed."

On the day preceding the operation, it is better to give the patient a smart purgative, and, after it has operated, to prescribe a dose of opium, in order to keep the bowels constipated for the two or three days following; then they may be opened with castor-oil. The operation, so far as Sir B. Brodie has seen, has been successful. In one case, however, that of a peculiarly nervous lady, death ensued from peritoneal and pleural inflammation and effusion.

It is difficult to pronounce on the quantum of danger that may follow operations on the rectum. We have seen a patient, with simple ulcer of the rectum, die in consequence of a mere manual examination. Inflammation was produced, and it extended to the loose cellular tissue immediately external to the muscular tissue; from this it spread to the cellular tissue external to the peritoneum, and so terminated in the destruction of the patient. In another case of ulceration of the rectum after dysentery, in which the contraction of the cicatrix gave rise to a species of stricture, we saw death ensue from the introduction of a bougie. The instrument did not penetrate the tunics, nor did it appear to have done any mischief to the gut; but it gave rise to inflammation and suppuration in the cellular tissue external to the bowel, and this extending to the cellular tissue of the pelvis, the patient, as we mentioned, died.

### *Treatment of Ulcer of the Rectum.*

This may or may not occur in con-

* Med. Gazette, April 4, 1835.

nexion with spasmodic contraction of the sphincter. When it does, it is necessary to divide the sphincter; when it does not, division, from above downwards, of the ulcer and the mucous membrane only, frequently effects a cure. If the patient is averse to an operation, the Ward's paste, given internally, and gentle aperients to regulate the bowels, may succeed. In one case, Sir B. Brodie cured the ulcer by suppositories of Ward's paste and soap.

Sir B. Brodie makes no mention of caustic. We have twice cured this kind of ulcer of the rectum by means of the nitrate of silver. This was applied at first every other, and afterwards every third day, and the bowels were kept open with castor-oil. We believe that the lunar caustic has been frequently successful in the hands of other surgeons; at least we have read, somewhere, accounts of its beneficial employment.

*Stricture of the Rectum.*

There is great reason to apprehend that rectum bougies are much abused. Many patients have bougies poked constantly into the rectum, who have no stricture of that gut. We have at present under our care a gentleman, who had been treated with long bougies for stricture of the rectum. When we saw him, there was no stricture of the bowel within the reach of the finger. From the history and symptoms of the case, we concluded that there was disease of the upper part of the intestinal canal. We recommended a total discontinuance of the use of the bougie, and general remedies, which we need not stop to specify. The patient has now ceased to use the bougie for some time, and his symptoms have improved rather than retrograded, a pretty good proof that our opinion, respecting the supposed stricture of the rectum was correct. The following sentiments of Sir B. Brodie on this head, should sink deep in the minds of surgeons.

"Strictures of the rectum are commonly situated in the lower part of the gut, within the reach of the finger. Are they ever situated higher up? I saw one case, where stricture of the rectum was about six inches above the anus; and I saw another case where there was stricture in the sigmoid flexure of the colon, and manifestly the consequence of a contracted cicatrix of an ulcer which had formerly existed at this part. Every now and then, also, I have heard from medical practitioners of my acquaintance, of a stricture of the upper portion of the rectum, or of the sigmoid flexure of the colon, having been discovered after death. *Such cases, however, you may be assured, are of very rare occurrence.* Inquire of anatomists who have been for many years teachers in the dissecting-room, or of surgeons who have witnessed a great number of examinations in the deadhouse of an hospital, and they will bear testimony to the correctness of what I have now stated.

Nevertheless, an opinion has of late years prevailed among some members of our profession, that a stricture high up in the rectum is a very frequent cause of constipation of the bowels; and I have known an almost incredible number of persons who have been treated on the supposition of their labouring under such a disease, by the introduction of long bougies into the bowel. The only evidence of the existence of a stricture in these cases has been, *first,* that there was obstinate costiveness; *secondly,* that a bougie introduced into the rectum could not be made to pass beyond a certain number of inches beyond the anus.

But what is the value of this evidence when compared with that which anatomy affords of the rarity of this kind of stricture? Are there not many causes of a costive state of the bowels besides mechanical obstruction? Will it be always easy, even in the most healthy rectum, to introduce a bougie more than a few inches into it? Although we call the lower bowel the *rectum,* you know very well that it is any thing but a straight gut. Three or four inches above the anus the rectum begins to make flexures, which increase as you trace it upwards, until they terminate in the sigmoid flexure of the colon. These flexures of the rectum differ in different individuals, and even in the same individual at different periods. When a bougie is introduced, be it small or large, it is certain that it will be stopped somewhere or another by one of these flexures; and nothing can be more unphilosophical than to conclude, because a bougie meets with an impediment at the distance of five or six, or eight or nine inches, that this is the result of an organic disease of the rectum, when the natural formation of the parts will sufficiently account for it.

But let us suppose that you actually meet with one of those rare cases in which there is a stricture in the upper part of the rectum; by what means are you to recognize the disease in the living person? Or, if you can recognize it, how can you know its exact situation? If the bougie can only be introduced to a certain distance, how are you to be certain that it is stopped by the stricture, and not by a fold of the bowel, or even by coming in contact with the sacrum?

Further than this, if you employ the force which you would suppose to be necessary to make the bougie penetrate through the stricture, is there no danger of it penetrating the tunics of the intestine instead? This last is no theoretical objection to the use of these long bougies in diseases of those parts, I will not say that I have seen the patients; but I have been informed on good authority of not less than seven or eight cases in which this frightful accident occurred, and the patients died in consequence.

Taking all these things into consideration, I advise you to lay it down for yourselves as a rule of practice, that you should not use bougies for stricture of the rectum, except where the stricture is within reach of the finger. If there be any exceptions to this rule, they are very rare indeed."

We repeat, that we recommend surgeons to lay the experience and advice of Sir Benjamin Brodie to heart, for they may be very well assured that it is much to the advantage of themselves and their patients to do so.

Sir B. Brodie's treatment of stricture of the rectum may be easily summed up. If good is to be done, it is at first. In the later stages of stricture of the rectum, ulceration of the stricture and of the contiguous gut—abscesses in the neighbouring cellular membrane—sinuses, &c. may have formed. We may then palliate, but the patient far too frequently dies. In the early stage, the careful use of the bougie—if the stricture is circular and cord-like, nicking it with the bistoire cachée, and dilatation with the bougie afterwards—proper attention to laxatives and diet, may effect a great deal of good. But in stricture of the rectum, as in stricture of the urethra, a *cure* is very seldom obtained, for the patient, to avoid relapse, must now and then pass a bougie for himself for the remainder of his life. If accumulations of fæces take place above the stricture, a gum catheter should be introduced through the stricture and into the fæces, and then warm water, or soap and water should be injected daily, to dissolve as far as possible and get rid of the accumulation. Sir B. Brodie has sometimes seen mercurial ointment smeared on lint, and the latter applied on the bougie, of benefit—and the irritability of the rectum is occasionally relieved in bad cases by the internal use of the decoction of the achillea mille folium. Two ounces should be added to a pint and a half of water, and this should be boiled down to a pint. The patient may take a wine-glass full three times daily.

### III. Mr. Green on the Employment of the Seton for Hydrocele.*

Those who are acquainted with the writings of Mr. Pott, are aware that he employed the seton for the radical cure of hydrocele. It was then a great improvement, but a greater, the injection of the tunica vaginalis, coming afterwards, the seton was abandoned.

Mr. Green would seem to be dissatisfied with the operation of injection, and he has been making experiments with the seton in order to ascertain if he could not regulate its effects, and find in it at once an effective and a mild remedy for hydrocele.

* St.Thomas's Hospital Reports.

Omitting a good many observations which contain nothing that would be novel to the greater part of our readers, we will extract at once Mr. Green's mode of procedure, and his own account of his success. It forms the termination of the lecture.

"After duly weighing all these important circumstances, it struck me that if a seton were carried through the tunica vaginalis, there would be a source of irritation sufficient to produce the required inflammation, and at the same time the opportunity given of regulating its degree, that is, that the seton might be allowed to remain till there were symptoms of such a degree of inflammation as is requisite for the change necessary to be produced in the tunic, and that this being effected the seton might then be withdrawn; and that the extraneous irritant being thus removed, it would have no further effect than was necessary either for the change of the surface of the membrane or for the obliteration of the tunic. It was with this view that the plan was adopted in the cases which I have cited.

The first case was indeed a partial failure, and the cause is evident; for the seton was withdrawn too early, in consequence of the pain he experienced after a very few hours. The operation was performed at one o'clock and the seton was withdrawn at ten, a period too short for the production of the requisite inflammation. Probably, indeed, the irritation tended to produce a quicker re-accumulation of the fluid. It is worth notice in this case, that the same individual should evince so much less disposition to inflammation in the second operation than in the first, although the second operation so quickly succeeded the first. It will be seen too that in this case some benefit might be attributed to the use of iodine, and perhaps you will not consider the following observations out of place.

A boy was brought to me a few days back, whom I had seen about two months since. I had recommended, although the boy was young, puncturing the tunic, for it was a hydrocele of large size with which he was afflicted. But there were some circumstances which induced the father to prefer it not being done at that time. In the meanwhile I ordered the *Unguentum Hydriodatis Potassæ,* according to Lugol's formula, to be rubbed on the scrotum daily. This was done during six weeks, and the fluid had become absorbed. I have not much faith, howver, in the application being generally useful in this respect; for, as far as my experience goes, local applications, in cases of hydrocele, are generally useless, except in very young persons. Hydrocele is by no means uncommon in children, and it is not necessary to have recourse to an operation in them: for in general it may be removed by a lotion (as that of *Liq. Ammon. Acet.* with spirit, or a solution of muriate of ammonia with vinegar), which is cold so as to produce a constriction of the scrotum, and at the same time stimulating in order to excite the vessels.

The second is a successful case, with the exception of the little suppuration in the cellular membrane.

The third completely answered my expectations.

In the fourth case suppuration took place in the cavity of the tunica vaginalis, which rendered it necessary that the tunic should be slit open in order to allow the escape of the purulent fluid.

The fifth case presents nothing remarkable; it was completely successful, the threads having been withdrawn after twenty-four hours.

The sixth case likewise has nothing remarkable in it, and we have only to draw attention to the successful result.

In the seventh case the success was not complete; but it appears that two previous operations by injection had entirely failed.

In the eighth case a second operation was required.

Now, generalising these facts, the results of the above and other cases, I may venture perhaps to say that the plan of treatment is well adapted to answer the end for which it was intended. In two instances indeed the operation failed from the want of a sufficient degree of inflammation, but which simply depended upon the insufficiency of the seton threads.

In another case there was a slight suppuration of the cellular membrane of the scrotum, which, however, only interfered with the rapidity of the cure, but was in no other way detrimental to the patient, nor prevented his speedy recovery. In another case, however, there was excessive inflammation and a suppurative process in the tunica vaginalis; and the possibility or probability of this occurrence is perhaps the most serious objection to the operation proposed which may be gathered from these cases. It might indeed raise in the mind a doubt on the principle itself of the operation. You introduce an extraneous body into the tunic, and you allow it to remain till inflammation is produced, and it is possible that the inflammatory action excited by extraneous bodies may tend to the suppurative instead of the adhesive form of inflammation. As, however, the result was only observed in one case, and as no such disposition was manifested in a number of cases, of which the success was perfect, we are perhaps warranted in drawing a conclusion generally in favour of the effects of the seton. Of course future cases (and as I shall continue to adopt the same plan of treatment such will not be wanting) will decide the point; but otherwise, in respect of having a mode of treatment enabling us to regulate the degree of inflammation, the plan here offered presents great advantages."

Thus we see that out of eight cases treated by the seton, two only were quite successful. One was doing well when the patient was dismissed, and the result left undetermined; a fourth was ultimately cured, but nearly two months elapsed, and some suppuration occurred in the cellular tissue of the scrotum. A fifth required two operations for the cure. In a sixth case, two operations were performed, and the patient was not ultimately cured. In a seventh case the seton was twice used, and this patient also was *not* cured—and finally, in another case, severe suppuration in the tunica vaginalis ensued. We must own that these results are any thing but satisfactory, and we venture to prophecy that the seton will be frequently found to do too much or too little—to produce only partial adhesion, or to give rise to suppuration. We anticipate little from the subsequent trials of Mr. Green, but we shall take care to lay the results before our readers.

Mr. Green observes that the seton is very applicable to the treatment of ganglions and bursæ. It is a dangerous remedy. We have twice seen death ensue from introducing a seton into the bursa situated over the inferior angle of the scapula. The surgeon should be cautious how he meddles with bursæ, in the way of exposing their cavity.

### IV. Mr. Travers on Absorption of the Cranium.

The St. Thomas's Hospital Reports contain a curious case, that formed the subject of a clinical lecture on the part of Mr. Travers. The case, greatly abridged, is this:—

Case. *Absorption of a Portion of the Cranium, following a contused Wound, with remarkable Symptoms of Cerebral Disorder.*

A postilion, æt. 46, was thrown from a gig, and struck his head with great violence upon the corner of a stone. This was in January, 1833. The scalp was cut and severely bruised, and he remained insensible for about half an hour, after which he recovered, and was able to walk to the surgeon's house to have the wound dressed. For a twelvemonth he continued tolerably well, occasionally, however, suffering from severe headache, giddiness, and general depression. This state was relieved by free purging, with abstemiousness and rest. During this period, however, he was generally engaged as a groom. At the termination of a twelvemonth he had a fit, was insensible for three or four days, and unable to resume his occupation for a period of six weeks. He passed the second year in much the same way as the preceding one. In February last (1835) he was engaged in the stable, and in the habit of driving the late Mr. James, surgeon, of Uxbridge, who observed that the man was much altered, having become forgetful and stupid. Towards the latter end of the same month, Mr.———, was called to him in the stable, and found him labouring under a well-marked epileptic fit. On examining the head, some irregularity and unevenness of the skull was detected. He remained insensible for four days, though apparently conscious at intervals, and within this period had two or three modified paroxysms, which were slight, and of short duration, but not a single well-marked epileptic fit as before. There was a prominence about the size of a shilling upon the side of the left parietal bone exquisitely painful, when touched, and when pressed upon, giving rise to a peculiar train of symptoms—viz. dimness of sight in both eyes, singing in the ears, deafness, giddiness, and a sense of intolerable weight upon the head. He had great restlessness—his pulse very weak and quick, but perfectly regular—his bowels obstinately costive, and the alvine secretions very dark and offensive. From this time he gradually, though very slowly recovered, to a certain point; but he still continued to have restless nights and frightful dreams, constant headache, the centre or precise seat of which he referred to the part above noted; his deafness increased; bowels very obstinate; pulse weak and slow; but he was still able to walk about, and the symptoms abated in a degree. At the expiration of two months from the commencement of this attack he was again seized in much the same way, except that the fits were more frequent; his bowels were always very much constipated, and the secretions in a very bad state; the local and general symptoms resembled those of the former attack so closely, as to render a description of them superfluous. He gradually became much emaciated, and continued to have fits, though slight, up to the time he was sent to the Hospital.

Loss of memory, and great despondency were prominent symptoms. On admission his case is described thus:—

Much emaciated—skin yellow—appetite bad—bowels torpid. He has a constant distressing heavy pain over the posterior superior part of the left temple, the seat of the blow, where a small circumscribed elevation is perceptible, and pressure on which produces increased pain. The pain also darts to the forehead and opposite temple, and sometimes becomes more severe, and he is then rather violent. He frequently talks incoherently, and only answers questions when spoken to loudly. Both motion and sensation on the right side of the body are impaired. His memory is much deranged, and he especially forgets dates and names, so that it is impossible to obtain from him any satisfactory account of his complaint. His mind is easily excited, and he scarcely sleeps at all. He gradually got worse—paralysis of the right side supervened—and the fæces were passed involuntarily.

On July 3, the operation of trephining was performed. On cutting through the integuments, which had no appearance of recent change, but were somewhat thickened, a perforation in the bone was discovered, about the size of the tip of the little finger, with a thin smooth border which extended through both tables of the skull, leaving the dura mater exposed. The greater part of the margin of this circular aperture was included in the application of the trephine: the under-surface of the bone and the dura mater were perfectly healthy, and had contracted no morbid adhesion, nor was there any thickening or detachment of the pericranium.

After the operation, he became much worse—was unconscious—lost his voice—was unable to protrude his tongue—and had total paralysis of the right side.

He was now put on a course of mercury, which salivated him severely. He had one fit afterwards, but gradually improved, and on the 15th of October was discharged cured.

This is a curious and instructive case. No doubt the absorption of the cranium was owing to an increased vascular action originally set up by the blow. The fits, paralysis, and so forth, shew that vascular action was not limited to the mere absorption of the cranium, which could, of itself, produce no such symptoms, but probably occasioned also some deposition about the arachnoid and pia mater, beneath the portion of the dura mater corresponding to the cranial aperture. The operation may have been of service by acting as an issue, and this, combined with the influence of the mercury, removed the inflammatory action and its consequences going on about the membranes, or beneath them. Such is the probable rationale of the case which we would offer.

Those who are anxious to learn the views of Mr. Travers, may refer to the Report itself. He ably illustrates by this case his peculiar doctrines of irritation.

### V. Mr. Guthrie on the Removal of Large Calculi.

The following very important case we have condensed from a clinical lecture delivered by Mr. Guthrie, and furnished to us by a friend and correspondent. The case is highly interesting.

*Lithotrity in the Female.*—The case of Mary Ken, who has lately left the hospital, has been one of great interest to us all. She came into it for the first time more than a year back, having then so large a stone in the bladder, that I feared it could not be removed by merely dilating the urethra, which I soon found to be the case, and decided upon breaking it up. She did not however submit patiently to the first trial, and the bladder contained, and could only be made to contain, but little water; I was therefore obliged to abandon the attempt after merely introducing the instrument, from finding that I could not persevere with safety, and she was so frightened that she could not be quiet, or refrain from falling into hysterics. From this time for several months, whenever the urethra was attempted to be dilated she had an attack of fever, apparently from fear, and became so generally ill that I despaired of her recovery, and indeed of her life. She went out of the hospital for a time to recruit her health if she could, but finding she was fit for nothing she returned, and after having fixed the day for operating many times and as often failed, she at last mustered courage to submit, saying she felt she could not live if she did not obtain relief, and this time she behaved magnanimously. The stone had greatly encreased in size since she first came into the hospital, and seemed to lie across the outlet of the pelvis, like the head of a child, whilst no urine could be retained in the bladder, the neck of which was dilated, so that the stone could be touched by a sound an inch in length. Being placed on a table arranged for the purpose (Heurteloup's), I divided the orifice of the urethra upwards with a straight bistoury introduced for one third of an inch within it. This admitted of that part being dilated much more easily, the peculiar structure at the very orifice not yielding readily, and often tearing rather than dilating. The urethra was now rapidly dilated by Mr. Weiss's dilator, until two fingers could be introduced, or a large pair of forceps, and I had several made with and without teeth for the purpose. None of them however could be opened so as to grasp the stone, and they merely rubbed or grated off small portions of the anterior part of it in the shape of sand or small grain. More than an hour had now elapsed, and nothing had been done of any consequence. Seeing that I never could open the forceps, I separated them like a midwifery forceps, and introduced one branch with some difficulty under the stone, and after several trials and with greater difficulty, the other branch was placed above it. They could not now however be made to lock at the hinge, so as to act efficiently upon the stone, which was so hard as to resist any moderate pressure, and that when another lithotrity instrument was screwed up upon them, they bent under the pressure until they met. It was therefore necessary to change them for a stronger pair, which was done, and at last the stone yielded in the middle, being apparently divided into two nearly equal parts. More than two hours had now elapsed, and some small pieces only had been got away. The urethra had also contracted, and the neck of the bladder also, and it became necessary to re-dilate these parts with the dilator, which was done in two or three minutes. I now separated Mr. Weiss's lithotrity instrument into two parts, and introducing the lower branch below one of the broken halves that of the right side, I drew it forward and fixed it against the pubes. The difficulty was now how to introduce the other branch without injuring the upper part of the urethra and neck of the bladder, but this was effected at last by placing a small scoop, such as is used for removing small pieces of stone on the point of it, and in this manner carrying it forwards very carefully. The instrument once fixed in this manner, acted well and broke the large piece of stone into many small ones by one crush. Several of these were now readily extracted, and the remaining half was also broken into two or three pieces by another proceeding of a similar kind, and four ounces and a half were extracted, together with the end of one of the first forceps tried, and which had been broken under the pressure of the screw-instrument on the branches. More than three hours had now elapsed, and Nature was nearly exhausted. I was therefore obliged to cease. She had taken several ounces of wine during the latter part of the time, and had borne the tedious and painful steps of the whole remarkably well. She was more exhausted however by the length of continuance of the operation than by the severity of the pain, as I took care the whole time to do nothing hastily, and to allow none of the instruments to do more than was intended. She was put to bed in the same room (the House Surgeon's), was diligently attended to, and had two grains of the muriate of morphia immediately, which quantity she was accustomed to take at night, and a grain from time to time until rest was procured.

It was quite delightful to find that she suffered little from so serious an operation; a great many small pieces of stone came away for several days, and a great deal of detritus, and one large piece was extracted by passing a probe behind it. Aware of the existence of another large piece in the bladder, she was anxious to have the operation repeated, and as soon as she was strong enough she fixed her day, and heroically submitted to it. I was now well prepared with instruments, the principal addition being a narrow sort of blunt gorget, which on the urethra being dilated, and one branch introduced, was passed in, and made to rest on the stone, so that the second branch of the screw lithotrity instrument could be readily run along it up to the stone, which was then crushed. The pieces thus broken up were brought away by a small but strong pair of forceps. There was great inconvenience experienced in using all these instruments, from the contracted state of the bladder, which grasped the larger pieces in such a way as to prevent their being easily seized or brought away. This was only overcome by opening the forceps well, and by that means dilating the bladder, when each piece was more easily grasped and brought away.—This operation was perfectly successful, and, by washing out the bladder from time to time, all the small pieces were brought away, and she has gone out perfectly free from calculus in the bladder. She is now quite fat, well, and a handsome young woman, free from pain, except a little in her back at times. She cannot however retain her water, and is obliged to wear an elastic bag, made for her liberally by Mr. Weiss, Jun. who furnished most of the instruments used in the different operations. The orifice of the bladder is contracted, so as to admit a common-sized female catheter less readily than usual, but it has not recovered its elasticity so as to shut the part close enough to prevent the urine escaping. She has improved on this point however, and I am in hopes she will continue to do so, and suffer less inconvenience. I have collected eight ounces and a half of calculus, principally of lithic acid, and she says herself she is sure as much more was lost—so as to make in all more than a pound by weight. How far this was the case I know not; but the stone certainly seemed to be by examination per vaginam as large as the head of a small child. The most valuable point for observation in this operation is the length of time the bladder suffered under the first attempt without sustaining any mischief; and I have satisfaction in thinking that this arose from the great care taken to let no instrument do more than it was intended it should do. I have reason to think that time is of much less consequence than is often supposed in the operation of lithotomy, and a man or a woman had better be an hour on the table than have the bladder either torn or materially injured. Some years ago I operated on a young man of nineteen, who had had a stone in his bladder from his birth, from which he had suffered excruciating torment, the bladder being always nearly empty, and incapable of retaining a couple of ounces of urine. I cut into the bladder and seized the stone in a minute, but it would not come out. I enlarged the opening with as little success;—the bladder greatly thickened, was firmly contracted round it, and processes from it appeared to adhere to the bladder. In 58 minutes it baffled all my efforts, my fore-finger resting upon its sharp processes, which seemed ready to tear the bladder before them. In this dilemma it struck me that I should never get it out, unless I could guard all these sharp points, and keep them away from the bladder as the stone came out. I sent for a very large pair of forceps, very much resembling a pair of firetongs, which I introduced, pushing the stone back before them. I then opened them forcibly, dilating the bladder as I did it, when the stone fell between the blades, and was removed in a moment. I cried out I have found out the use of the tongs, and very happy I was to do so. The stone looked exactly like a piece of coral rock, and was quite as irregular. It is an unique specimen of oxalate of lime. The man was bled the same evening, and never had a bad symptom afterwards, nor a return of his disease. The opening in the bladder in this case was as large as could well be made with safety and certainty, and far exceeded the boundaries of the prostate gland. I attributed his safe and speedy recovery to the care taken to do no violence to the bladder beyond what was always intended to be done. I might perhaps have pulled the stone out within the first few minutes, but I should have lost my patient.

